# Waking immune-resistant tumors with neddylation

**DOI:** 10.1172/JCI167894

**Published:** 2023-02-15

**Authors:** Kristin Huntoon, Wen Jiang, Betty Y.S. Kim

**Affiliations:** 1 Department of Neurosurgery and; 2 Department of Radiation Oncology, The University of Texas MD Anderson Cancer Center, Houston, Texas, USA.

## Abstract

The CD47/signal regulatory protein α (SIRPα) axis, which functions as an inhibitory phagocytosis checkpoint, also serves as a key mediator in cancer immune evasion. Many cancers, including colorectal cancer (CRC), exploit the expression of CD47 to escape phagocytic clearance and activate the innate immune system. Previous work has indicated that distinct paradigms of posttranslational modifications mediate the regulatory mechanisms of the CD47/SIRPα axis. In this issue of the *JCI*, Li et al. show that neddylation, a ubiquitin-like modification, inactivates Src homology region 2–containing protein tyrosine phosphatase 2 (SHP2), a downstream target of this pathway. They further show that inhibition of SHP2 sensitizes CRC cells to immunotherapies to which they were previously resistant. Collectively, the results underscore the need for cotargeting SHP2 and immune checkpoints (e.g., programmed death 1 [PD1]) in CRC and possibly other immunotherapy-resistant tumors.

## Immunosuppressive tumor microenvironment

Use of immune checkpoint inhibitors targeting tumor-infiltrating lymphocytes is increasing, but primary resistance to one widely used such inhibitor, anti–programmed death 1 (anti-PD1), is common, affecting up to 60% of patients with some types of cancer (1). This resistance is thought to result from an immunosuppressive environment within the tumor. Macrophages, a major component of innate immunity, are widely acknowledged as one of the central suppressive cell populations within the tumor microenvironment; depleting these cells under several therapeutic conditions can unleash T cell responses (2). Thus, controlling immune suppression requires inhibiting this suppressive activity and polarizing these macrophages so that they engulf tumor cells (3). The phagocytosis of tumor cells by macrophages relies on a balance between pro- and antiengulfment signals that are present on the surfaces of target cells (3). Thus, harnessing these signals is a promising strategy for cancer immunotherapy (4).

## The CD47/SIRPα axis

The balance of phagocytosis by antigen-presenting cells (APCs) is governed by a set of cell-surface markers that help the APCs to recognize cells as self or non-self. One such transmembrane protein is CD47, which imparts what is called the “don’t eat me” signal and is expressed on the surface of various cell types. CD47 binds to the transmembrane protein signal regulatory protein α (SIRPα) on myeloid cells (particularly macrophages) to form the CD47/SIRPα signaling complex (5). SIRPα on macrophages binds with CD47 to resist proengulfment signals. Unfortunately, malignant cells can take advantage of this intrinsic pathway by overexpressing CD47 to avoid being cleared by APCs (6). However, numerous studies indicate that administration of agents that block the CD47/SIRPα phagocytosis checkpoint leads to improved tumor clearance in vivo (7). Disruption of the CD47/SIRPα axis has shown remarkable antitumor effectiveness in recent clinical trials, furthering the rationale for engaging this pathway (8, 9)

Binding between CD47 and SIRPα results in the phosphorylation of two immunoreceptor tyrosine-based inhibitory motif (ITIMs) by Src family kinases, which are required for downstream signaling (10). The phosphorylation of the SIRPα cytoplasmic region results in recruitment of the nonreceptor protein tyrosine phosphatases Src homology region 2–containing protein tyrosine phosphatase 1 (SHP1) and SHP2 for signal transduction (11). However, some recent studies have noted that posttranslational modifications may have regulatory activity that further dictates activation of the CD47/SIRPα pathway (12, 13).

**Figure 1:**
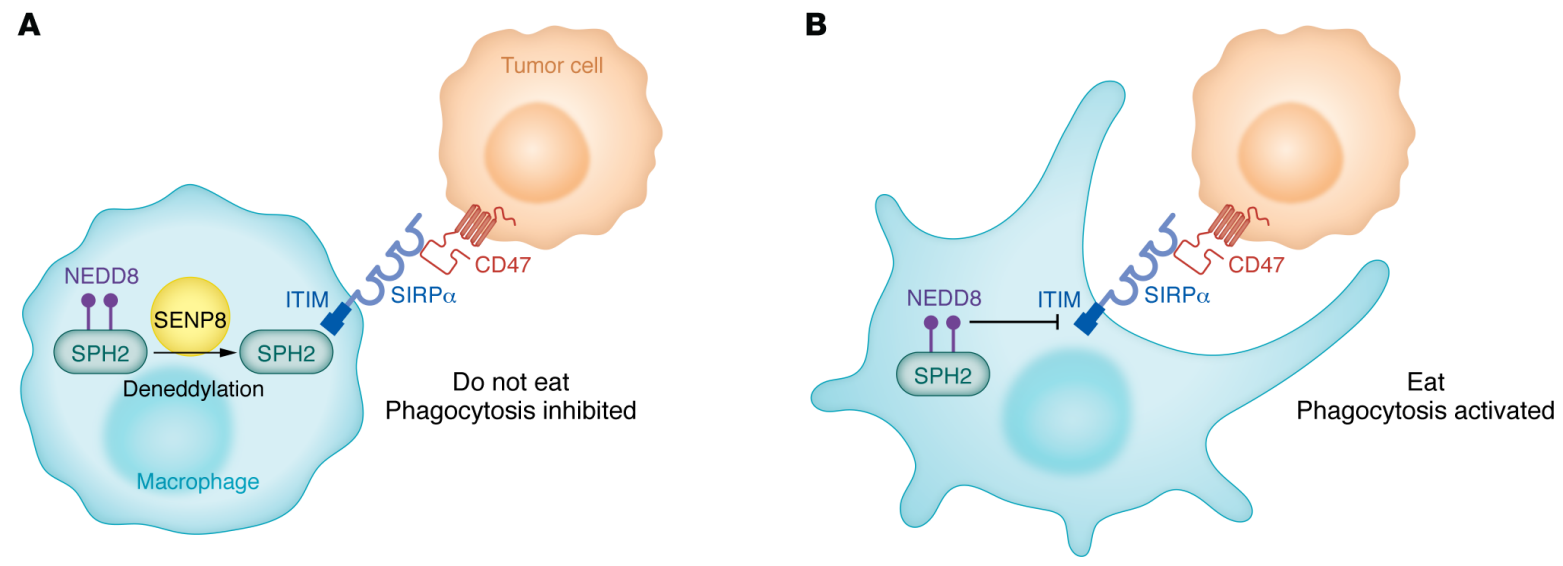
A proposed mechanism of action of the CD47/SIRPα/SPH2 signaling complex involves neddylation. (**A**) SIRPα is a transmembrane protein that contains three Ig-like domains in its extracellular region and two tyrosine phosphorylation sites within its cytoplasmic region. CD47 on tumor cells binds SIRPα on macrophages, promoting tyrosine phosphorylation of SIRPα and its subsequent binding to protein tyrosine phosphatases. This pathway is mediated by the deneddylation of SHP2 via SENP8, which activates the SIRPα-associated phosphatases. Specifically, SIRPα’s cytoplasmic ITIM facilitates this signaling sequence,resulting in the inhibition of phagocytosis (operating as a “do-not-eat-me” signal). (**B**) When SHP2 is neddylated, it cannot bind to the ITIM of SIRPα and thus promotes phagocytosis of the tumor cell.

Neddylation, a posttranslational mechanism that resembles ubiquitin-like modifications, refers to the conjugation of NEDD8 to a specific substrate (14). Neddylation can occur with key substrates, such as cGAS and Myd88 in macrophages, further supporting the premise that this mechanism can influence the tumor immune microenvironment (15, 16). In this issue of the *JCI*, Li and coauthors found that CD47/SIRPα signaling triggered substrate deneddylation in colorectal tumor–infiltrating macrophages (17). They first showed that the process of neddylation and NEDD8 expression were increased in various cancer types in SIRPα^+^ tumor-infiltrating macrophages. Next, they verified the findings by analyzing NEDD8 expression in tumor-infiltrating macrophages derived from colorectal cancer (CRC) mouse models treated with CD47 blockade or a control agent. Confirmation of the interplay between the CD47/SIRPα pathway and NEDD8 was substantiated by decreased deneddylation in CD68^+^ macrophages from tumor tissue organoids treated with anti-CD47 antibody. Li and colleagues systematically showed that the conjugation of NEDD8 at K358 and K364 of SHP2 was necessary for preserving its autoinhibitory conformation (Figure 1). They further demonstrated that SHP2 deneddylation was mediated by SUMO/sentrin-specific protease family member 8 (SENP8), resulting in its recruitment to SIRPα and consequent activation of signals vital for inhibiting macrophage phagocytosis. Neddylation had the exact opposite effect, that is, it induced SHP2 inactivation and potentiated the effect of immunotherapy in vivo. These investigators further confirmed that the activity of SHP2 in tumor-infiltrating macrophages from patients with CRC was stronger than the SHP2 activity in paired unaffected adjacent tissues, suggesting that SHP2 neddylation is reduced in CRC. They went on to show a potential correlation between the CD47/SIRPα axis in macrophages and prognosis for patients with CRC. With this knowledge, the authors next showed that the use of allosteric SHP2 inhibitors that prevent SHP2 from interacting with SIRPα led to synergistic responses to immunotherapy by disrupting the immunosuppressive CRC microenvironment in vivo, resulting in robust antitumor benefit as well as substantially reduced liver metastasis (17).

## Clinical implications of 

## SHP2 inhibition

SHP2 has emerged as an integrator of growth and differentiation signals from receptor tyrosine kinases into the RAS/mitogen-activated protein kinase cascade and cytokine receptor signaling (18). SHP2 is also a convergent node for several signaling pathways within immune cells and cancer cells and has the distinction of being the only protein tyrosine phosphatase designated as an oncogene (19). As discussed by Li et al., SHP2 acts as a leverage point in the CD47/SIRPα axis in macrophages by inhibiting intracellular signaling and thereby decreasing phagocytosis activity in these cells (17). However, SHP2 also binds to colony-stimulating factor-1 receptor (CSF-1R), part of a key axis in cellular survival, proliferation, and macrophage reprogramming, further supporting the notion that targeting SHP2 can promote antitumor immunity. Importantly, SHP2 plays another role in T cell function by binding to immune-inhibitory receptors responsible for regulation of T cell activation (e.g., PD1, cytotoxic T lymphocyte–associated protein 4 [CTLA4], T cell immunoglobulin and ITIM domain [TIGIT], B and T lymphocyte attenuator [BTLA], and others). In these pathways, SHP2 prevents activation of the PI3K/protein kinase B (also known as AKT) pathway, thus resulting in inhibition of T cell function and promotion of T cell anergy (20).

Several SHP2 inhibitors are already being studied in preclinical models of various types of hematologic and solid cancer (20), and several phase I clinical trials are underway in which SHP2 inhibitors are used alone or as part of combinatorial therapies, primarily for solid tumors (21).

## Conclusion

Li et al. showed that in macrophages, SHP2 is constitutively neddylated, but in the tumor microenvironment of CRC, the activation of macrophage SIRPα, via tumor cell CD47, results in SHP2 deneddylation by SENP8, which subsequently inhibits the phagocytic activity of tumor-infiltrating macrophages. However, an SHP2 allosteric inhibitor in combination with immunotherapy agents such as anti-PD1 restored tumor clearance (17). Although the experiments in Li et al. focused on CRC, they support the rationale for emerging trials in which SHP2 allosteric inhibitors are used with immune checkpoint inhibitors for other, traditionally immunosuppressive tumor types. Future trials that include clinical specimen analyses could guide clinical applications in the event that the antitumor effectiveness of SHP2 inhibitors is limited. Other targets along this CD47/SIRPα/SHP2 pathway have the potential to provide effective treatments for patients with CRC and other immunotherapy-resistant tumors.

## Version 1 02/15/2023

Electronic publication
